# Advancements in the application of reporter gene cell lines in bioactivity evaluation of biological products

**DOI:** 10.1186/s40643-025-00932-2

**Published:** 2025-09-24

**Authors:** Kaijuan Yi, Can Wang, Huili Lu

**Affiliations:** 1 Engineering Research Center of Cell and Therapeutic Antibody, Ministry of Education, School of Pharmacy, Shanghai Jiao Tong University, Shanghai, 200240 China; 2 NMPA Key Laboratory for Quality Control of Therapeutic Monoclonal Antibodies, Shanghai Institute for Food and Drug Control, Shanghai, 201203 China

**Keywords:** Biological activity, Reporter gene assay, Stable cell line, CRISPR/Cas9, Targeted integration

## Abstract

The assessment of biological product activity is a key aspect of quality control. Currently, in vitro assays serve as the primarily method employed by both companies and regulatory agencies to evaluate biological activity. Reporter Gene Assay (RGA) is a technique that investigates gene expression regulation and cellular signal transduction pathway activation through easily detectable reporter genes. RGA is highly dependent on drug mechanisms, offering high accuracy and precision, and has gained increasing recognition. The utilization of alternative analytical methods based on RGA have emerged as a prevailing trend, with a growing number of antibody drugs adopting corresponding RGA-based quality control approaches. Establishing stable expressing cell lines is essential to ensure the stability, reliability, and consistency of assays across diverse conditions when employing RGA techniques. CRISPR/Cas9 gene editing technology mediated site-specific gene integration allows for rapid and precise insertion of exogenous genes into specific genomic loci and enables the efficient construction of stable RGA cell lines, which would significantly propel the advancement of biological activity evaluation methods.

## Classification of biological activity testing methods for biologics

Biological products serve as a critical driving force for the biopharmaceutical industry. Quality control is the key process to guarantee the efficacy of biological products, particularly, biological activity assays are crucial for evaluating drug potency and content (Register et al. 2021). To confirm the quality attributes of biological products, the ICH (European Medicines Agency (EMA), 1999) and other regulatory guidelines mandate the establishment of various activity analytical methods to comprehensively assess their biological activity.

Currently, the evaluation of the bioactivity of biological products mainly relies on the establishment of in vitro cell evaluation models to simulate their mechanisms of action, generating reliable dose–response processes, and assessing their bioactivity through comparison with known active reference substances. Common biological activity methods are divided into three types: cell-based, transgenic cell-based, and new technology-based methods. The classification and advantages, and disadvantages of each method are detailed in Table 1 (Singh et al. 2024). The comparison of key performance metrics for biological detection methods are summarized in Table 2.

**Table 1:** Comparison of Biological Detection Methods for Monoclonal Antibody Drugs ^\* MERGEFORMAT (^Register et al. 2021)

Classification	Detection method	Mechanism	Advantages	Disadvantages	Example
Cell-based Activity methods	Cell Proliferation inhibition assay	Cell proliferation cell proliferation inhibition based on cellular mechanisms	Easy operation, High specificity, Good reproducibility, High accuracy	Requires optimization of staining solution, Selection of statistical model	Avastin (VEGF mAb), Herceptin (Her2 mAb)
	Cytotoxicity assay	Cell apoptosis, Caspase activation based on cellular mechanisms	Easy operation, High specificity, Good reproducibility, High accuracy	Requires optimization of staining solution, Selection of appropriate statistical model	Humira (TNF-α mAb)
	CDC	CDC action based on cellular mechanisms	Easy operation, High specificity, Good reproducibility, High accuracy	Complex complement source, Different formulations, Multiple components, Heat instable and prone to inactivation	Rituximab (CD20 mAb)
	ADCC	ADCC action based on cellular mechanisms	Easy operation, High specificity, Good reproducibility, High accuracy	Cell separation and culture are difficult, High variability, Cumbersome operation, High background value	Rituximab (CD20 mAb)
	ELISA	ELISA method based on cellular mechanisms	High sensitivity, Strong specificity	Selection of appropriate ELISA detection indicators and sensitive evaluation methods	Yervoy (CTLA-4 mAb)
Transgenic cell-based methods	Reporter gene assay	Constructs transgenic cell lines expressing luciferase reporter gene based on drug mechanism of action	High specificity, Easy operation, High sensitivity, Good stability, High throughput screening, High efficiency, Easy gene editing, Reproducibility, Easy gene regulation, Multifunctionality, Drug safety assessment, Assists in drug mechanism research	More complex cell line construction, Method validation required, high cost	Opdivo/Keytruda, Herceptin, Avastin, Zolgensma, Luxturna, Kymriah, Fabrazyme
New technology-based activity methods	SPR potency	Surface Plasmon Resonance	Reflects affinity and kinetic parameters of molecular interactions	Expensive instruments	Antigen–antibody binding activity, antibody-receptor binding activity
	HTRF	Homogeneous Time-Resolved Fluorescence	High sensitivity	Reflects antibody binding activity	IGF-1R mAb
	Alpha technology	Alpha Technology	High sensitivity	Reflects antibody binding activity	HGF mAb
	Fluorescent dye labeling method	Fluorescent Dye Labeling	Simple method, High selectivity, High sensitivity, Low sample usage	Selection of appropriate fluorescent dye for cell/molecule labeling	Antibody activity determination related to cell viability

**Table 2:** Comparison of key performance metrics for biological detection methods (Piede et al. 2023; Yu and Yang 2017)

Classification	Detection method	Limit of detection (LOD)	Dynamic range	Intra-batch CV (%)	Inter-batch CV (%)
Cell-based activity methods	Cell proliferation Inhibition assay	~ 10–9–10–12 M	PBMC:MSC ratio of 1:1 to 1:0.1	Below 10%	Below 15%
	Cytotoxicity assay	~ 100 cells per test well	10–90% cell death	Below 10%	Below 15%
	CDC	~ 10^–6^ M	10–90% cell death	Below 15%	Below 20%
	ADCC	~ 10^–7^ M	20–90% cell death	Below 15%	Below 20%
	ELISA	~ 10^–9^–10^–12^ M	Wide, typically 10^2^–10^5^	~ 2–10	~ 5–15
Transgenic cell-based methods	Reporter gene assay	~ 10^–12^ M	10^2^–10^6^ relative light units	Below 10%	Below 15%
New technology-based activity methods	SPR	~ 10^–9^ M	Wide, typically 10^4^—10^6^	~ 1–5	~ 5–10
	HTRF	~ 10^–12^ M	Moderate, typically 10^2^–10^4^	~ 2–8	~ 5–12
	Alpha technology	~ 10^–11^ M	Moderate, typically 10^2^–10^4^	~ 3–10	~ 6–15
	Fluorescent dye labeling method	~ 10^–8^ M	Moderate, typically 10^2^–10^4^	~ 2–10	~ 5–15

### Cell-based bioassay methods

Cell-based bioassay methods utilizes in vitro cell culture technology to evaluate the bioactivity of products, mainly include the following: (a) Cell Proliferation Inhibition Method: For monoclonal antibodies targeting specific proliferation-related targets such as VEGF, HER2, or EGFR (Register et al. 2021), their activity can be assessed through cell growth inhibition. (b) Cytotoxicity Method: For drugs targeting cell-killing related receptors, such as TNF-α receptor-antibody fusion proteins, they can be evaluated by detecting cell apoptosis (Register et al. 2021). (c) complement-dependent cytotoxicity (CDC): For monoclonal antibodies whose activity depends on CDC function, such as CD20 or CD52 mAb, CDC activity can be measured (Register et al. 2021) (d) antibody-dependent cell-mediated cytotoxicity (ADCC): This is applicable for assessing the ADCC immune efficacy mediated by the Fc region of mAb drugs using PBMC or NK as effector cells (Wang et al. 2018^) (^Zhu et al. 2023). (e) ELISA: This is available for detecting cytokines released by target cells after co-incubation with antibodies and activators to evaluate the biological function of the antibodies (Gupta 2025).

### Transgenic cell-based biological activity assay method

Many biologics lack sensitive cell lines or easily measurable cellular effects during development, which limits the effective assessment of their biological activity. To address these challenges, researchers create transgenic cell lines utilizing genetic engineering techniques to introduce specific genes (usually reporter genes) into cells (Lei et al. 2023; Gao et al. 2022). Reporter genes (RG) are a class of genes that can be expressed within cells and produce measurable signals. Common reporter genes include luciferase and β-galactosidase. Transgenic reporter gene cell lines provide a stable and reproducible experimental system, helping to reduce dependence on primary cells, minimizing variability, and enhancing the accuracy and reliability of experiments.

The design of transgenic cell lines needs to be based on the mechanism of action of the drug. For example, if a drug works by activating a specific signaling pathway, a cell line that includes a reporter gene related to that pathway can be designed. When the drug interacts with the transgenic cell line, it will activate or inhibit the expression of the reporter gene, and then produce measurable signals such as fluorescence or luminescence. This analytical method allows for high-throughput screening to evaluate the activity of drug candidates, which is crucial during the drug screening and development stage. Several global mAbs have utilized transgenic cell lines with reporter genes for biological activity assays during their development process, demonstrating the effectiveness and universality of this approach (Wang et al. 2020).

### New technology-based biological activity assay method

The new technology-based biological activity assay method mainly includes four categories: (a) Surface Plasmon Resonance (SPR): SPR does not require labeling, it evaluates the binding activity between antibodies and antigens by detecting optical effects at the interface of media with different refractive index (Capelli et al. 2023). (b) Homogeneous Time-Resolved Fluorescence (HTRF): HTRF is a method used to detect analytes in pure liquid samples. It combines Fluorescence Resonance Energy Transfer (FRET) with Time-Resolved Fluorescence (TRF), offering advantages such as easy operation and high sensitivity (Liang and Zhang 2022). (c) Alpha Technology: This technique uses proximity-based donor and acceptor beads, where the donor bead is conjugated with the antigen and the acceptor bead with the antibody. Upon close proximity, a chemical reaction is rapidly triggered, producing a light signal (Zeng et al. 2020), thereby measuring the biological activity of the antibody. (d) Fluorescent Dye Labeling: This method involves covalently binding or adsorbing fluorescently labeled chemicals (known as fluorophores) to specific groups of the molecules in the study. Fluorescence is then utilized to obtain molecular information (Grossenbacher et al. 2022; Singh and Roy-Chowdhuri 2016; Blay et al. 2020).

M means nanomolar.

## Reporter gene assays

Due to many biologic drugs lacking cell lines with strong responsiveness or easy detectability, reporter gene assays (RGAs) are gaining increasing recognition in the field of biopharmaceutical quality control due to their close correlation with the mechanisms of action of biological products and their high precision and accuracy (Blay et al. 2020). The activity assessment methods based on this approach have become an alternative trend.

### The molecular biology principles of RGAs

Common reporter genes include a regulatory response element and the reporter gene itself. The regulatory response element is responsible for controlling the expression of the sequence, while the reporter gene encodes a protein or enzyme that is easily detectable and controlled by the response element (Wang et al. 2020). RGAs integrate specific reporter genes into host cells through molecular technology. Upon stimulation by signaling molecules, these genes are activated by specific regulatory sequences and express products that can either directly emit a signal or indirectly generate a signal. Therefore, RGAs enable highly sensitive tracking and measurement of gene-related intracellular signaling transduction processes (Wang et al. 2020; Piede et al. 2023).

Currently, various types of reporter genes have been developed, with commonly used categories including chloramphenicol acetyltransferase gene (CAT), β-galactosidase gene, luciferase gene, β-lactamase gene, among others. The advantages and disadvantages of these reporter genes are detailed in Table 3. The luciferase is a type of enzyme that can emit fluorescence naturally by catalyzing specific substrates to produce spontaneous fluorescent signals. Due to its easy detection and high sensitivity, luciferase is one of the most commonly used enzymes in reporter gene assays for biological activity assays. The most common luciferases include Renilla luciferase and Firefly luciferase (Blay et al. 2020; Yu and Yang 2017).

**Table 3:** Classification and Comparison of Reporter Genes (Blay et al. 2020; Piede et al. 2023)

Reporter gene category	Advantages	Disadvantages
Chloramphenicol acetyltransferase (CAT) Gene	More stable expression products, very low background in eukaryotic cells, good reproducibility, high sensitivity	Relatively lower sensitivity, high cost of isotopes and thin-layer chromatography systems, safety issues for operators and environment
β-Galactosidase gene	Easy detection, widely used for in situ chromosome staining, relatively stable, no need for radioactive elements	High endogenous activity in certain cells, lower sensitivity for non-chemiluminescent detection
Luciferase gene	Convenient and quick with high sensitivity, large linear range, very low background, relatively simple analysis methods	Requires high sensitivity luminometers, relatively unstable proteins
β-Lactamase gene	Easy to use colorimetric assay for secreted reporter proteins, fluorescent gene provides high sensitivity	Expensive detection equipment, unable to perform in situ analysis

Additionally, the transformative advancements through the integration of biophysical techniques and microfluidic technologies are developed. The dual-reporter systems such as BRET/FRET use BRET (Bioluminescence Resonance Energy Transfer) and FRET (Fluorescence Resonance Energy Transfer) to hybrid these technologies. BRET technology has been applied to develop biosensors for metabolites and enzyme activities. FRET biosensors are used to study the pharmacology of G protein-coupled receptors (GPCRs), aiding in the deorphanization and investigation of novel receptors. The hybrid BRET-FRET technology study a hyBRET biosensors through transform intramolecular FRET biosensors into BRET-FRET hybrid biosensors, using a bioluminescent donor to excite a fluorescent acceptor, while FRET requires external light for excitation. This dual-reporter system can retain the advantages of FRET biosensors while leveraging the strengths of BRET technology. The transgenic mice expressing hyBRET biosensors enable non-invasive visualization of pharmacodynamics, accelerating drug development (Komatsu et al. 2018). Microfluidics-integrated reporter assays are a cutting-edge analytical technology combining microfluidics with reporter assays. They operate by incorporating reporter molecules into microfluidic systems, where target molecules react with these reporters to produce detectable signals. The technology is applied in pathogen detection, drug screening, environmental monitoring, biomedical research, and other fields, etc. (Huang et al. 2025).

A comprehensive summary table mapping major biologic target classes to validated reporter gene assay (RGA) readouts is provided in Table 4 (Li et al. 2021; Baah et al. 2021). RGA readouts are typically designed to reflect downstream gene expression changes (e.g., NF-κB activation drives luciferase expression via a promoter containing NF-κB binding sites). Most readouts listed are validated in peer-reviewed literature or clinical trial assays, with examples including luciferase-based assays in FDA-approved drug screening protocols.

**Table 4:** A summary table about to validated Reporter Gene Assay (RGA) readouts (Li et al. 2021; Baah et al. 2021)

Biologic class	Target pathway/molecule	Validated RGA readout
Monoclonal antibodies	TNF-α (Tumor necrosis factor-α)	Luciferase reporter for NF-κB activation
	PD-1/PD-L1	GFP reporter for T-cell activation
Cytokines	IFN-γ (Interferon-γ)	Luciferase reporter for JAK-STAT pathway
	IL-6 (Interleukin-6)	SEAP (Secreted Alkaline Phosphatase) reporter for STAT3 activation
Fusion proteins	TGF-β (Transforming Growth Factor-β)	Luciferase reporter for SMAD2/3 signaling
	VEGF (vascular endothelial growth factor)	RFP (Red fluorescent protein) reporter for PI3K-AKT pathway
Small molecule inhibitors	EGFR (Epidermal growth factor receptor)	Luciferase reporter for MAPK/ERK pathway
	NF-κB (nuclear factor kappa-light-chain-enhancer of activated B cells)	β-Galactosidase reporter for NF-κB nuclear translocation
siRNA/miRNA therapeutics	TLR4 (toll-like receptor 4)	Luciferase reporter for NF-κB and IRF3 pathways
	Oncogenic miRNAs (e.g., miR-21)	GFP reporter for PTEN/AKT pathway restoration
ADCs	HER2 (human epidermal growth factor receptor 2)	Luciferase reporter for MAPK/ERK pathway
	Trop-2 (trophoblast cell surface antigen 2)	SEAP reporter for PI3K-AKT pathway
	CD19 (cluster of differentiation 19)	GFP reporter for NF-κB activation

### Application of RGAs in Biological Activity Evaluation

RGAs have been applied to evaluate the biological activity of various biologics, including but not limited to cytokines, hormones, and antibody drugs (such as anti-cytokine/cytokine receptor antibodies, tumor-associated antigen antibodies, and immune checkpoint blocking antibodies) (Loughran et al. 2025; Calabretta and Michelini 2024). The Chinese National Institutes for Food and Drug Control (NIFDC) is at the forefront of employing reporter gene technology and has established a transgenic activity measurement model system that is efficient, rapid, accurate, and non-animal safety principle-compliant transgenic activity (Register et al. 2021). For instance, Liu et al. at NIFDC (Chun-yu et al. 2019) developed an ADCC detection method for Trastuzumab (Peng et al. 2024) based on the reporter gene method. Although one of the mechanisms of action for Trastuzumab is through ADCC (Mandó et al. 2021), the biological activity evaluation of this mAb has primarily focused on cell proliferation ability and the antigen–antibody binding capacity assessed by ELISA, with relatively less research on ADCC. The authors used a genetically modified Jurkat cell line to pioneer an accurate and rapid method for measuring the ADCC activity of anti-HER2 antibodies (Chun-yu et al. 2019; Liu et al. 2021). This cell line carries a luciferase reporter gene driven by an NFAT (nuclear factor of activated T cells) promoter element, which drives the expression of luciferase. By utilizing the BioGloTM Luciferase Assay System (a detection system based on luciferase activity), the intracellular luciferase activity can be quantitatively detected. A well-defined dose-responsive S-shaped curve can be obtained after appropriate dilute Anti-HER2 mAb. Analytical method validation demonstrated that this analytical method has high reproducibility and low variability, and can serve as a a standard method for evaluating the ADCC biological activity of anti-HER2 mAb (Chun-yu et al. 2019; Liu et al. 2021).

Additional, reporter-gene assays are instrumental in viral vectors, CAR-T-cell products, and nucleic-acid therapeutics. They enable researchers to monitor infection, assess efficacy, track distribution, evaluate activation, and optimize drug design. A study (Golm et al. 2023) used a fluorescent protein reporter gene in an AAV vector to track viral replication in real time, revealing the dynamics of viral gene expression. A study linked a luciferase reporter gene to activation-related elements in CAR-T cells. They found (Morath et al. 2025) that specific tumor antigens triggered significant luciferase expression, indicating CAR-T cell activation and guiding the design of more effective CAR constructs. Researchers (Shapiro et al. 2025) tested different promoter sequences linked to a luciferase reporter gene for mRNA constructs. They identified a synthetic promoter that increased luciferase expression by fivefold, optimizing mRNA design for higher efficacy.

Additionally, the reporter gene method has also been used to evaluate the biological activity of bispecific antibodies (Chen et al. 2021). Catumaxomab injection is the first EpCAMxCD3 bispecific antibody approved by the EMA. The traditional method for evaluating its biological activity is based on PBMC cytotoxicity assays. This method is cumbersome, time-consuming, and relies on PBMCs provided by blood donors, which introduces individual variability (Ma et al. 2021). In light of these limitations, Zhang et al. (Hong-Mei et al. 2021) developed a method based on passaged cells and reporter genes. They selected the HCT116 cell line as target cells and HuT78 cells as effector cells. The plasmid DNA pGL4.51[Luc2/CMV/Neo] was introduced into HCT116 cells to screen for clonal HCT116-Luc cell lines with high expression of luciferase. In the presence of the anti-EpCAM xCD3 bispecific antibody, HuT78 cells, and transgenic HCT116 cells, the overall expression of luciferase was positively correlated with the number of transgenic HCT116 cells and exhibited antibody concentration dependence. By measuring the intensity of luciferase activity and fitting the dose–response curve with a four-parameter model, the relative biological activity of the product can be calculated (Hong-Mei et al. 2021).

Overall, the reporter gene method develops the regulation of gene expression and the activation of cell signaling pathways by utilizing easily detectable reporter genes. Due to its high sensitivity, ease of quantification, convenient operation, short cycle, low cost, suitability for high-throughput screening, and ease of detection, this method has been widely applied and developed in the biological activity assessment of biologics. However, there are still some challenges in the establishment of this method, such as transfection efficiency, stable expression of the introduced gene, and clonality of recombinant cell lines. These are critical points that require careful monitoring and resolution (Wang et al. 2020; Blay et al. 2020).

## RGA cell lines construction and CRISPR/Cas9 mediated site-specific integration

To ensure the consistency of RGAs biological activity analytical methods in different environments, it is crucial to develop stable RGAs cell lines from the same source or using similar methods. However, the currently available RGAs cell lines are relatively few, making it difficult to meet practical needs. Moreover, the currently available commercial cell lines may have issues such as genotypic instability. Therefore, a stable and reliable RGAs transgenic cell line is urgently developed and constructed, which has great significance for enhancing the application level of RGAs (Wang et al. 2020; Liu et al. 2021).

### Traditional cell lines construction methods

Traditional methods of constructing cell lines mainly rely on the random insertion and screening process of exogenous genes (Le et al. 2015). First, vectors carrying the target gene are introduced into host cells through transfection methods such as retroviruses, electroporation, calcium phosphate precipitation, or liposomes. Once the DNA enters the cell nucleus, it will be randomly inserted into the host chromosomes (Majumdar et al. 2025) (Zeh et al. 2024). Subsequently, a selection drug like puromycin is used to select cell populations that correctly and highly express the target gene, followed by multiple rounds of pressure screening and identification to obtain monoclonal cell lines (Zeh et al. 2024). However, this method involves a large workload and high complexity, and the genetic stability of the obtained monoclonal cells is poor. Due to potential “position effects” (i.e., gene expression variability caused by the uncertainty of the insertion site of the exogenous gene), the expression level of the target gene may decrease over time, resulting in an impairment of the performance of the selected cell lines during continuous culture (Li et al. 2018; Sahoo et al. 2020). Therefore, using site-specific integration techniques to integrate the target gene into stable and transcriptionally active genomic loci is an effective means to obtain highly expressing stable cell lines. Achieving high-efficiency site-specific integration is crucial for developing stable RGAs cell lines. The CRISPR/Cas9 gene-editing technology can efficiently achieve site-specific integration of exogenous genes, integrating the target gene into specific regions of the cell chromosomes, thereby achieving stable expression (Hub et al. 2024; Azeez et al. 2024).

### Application of CRISPR/Cas9-mediated targeted integration technology in the construction of RGA cell lines

**Fig. 1:**
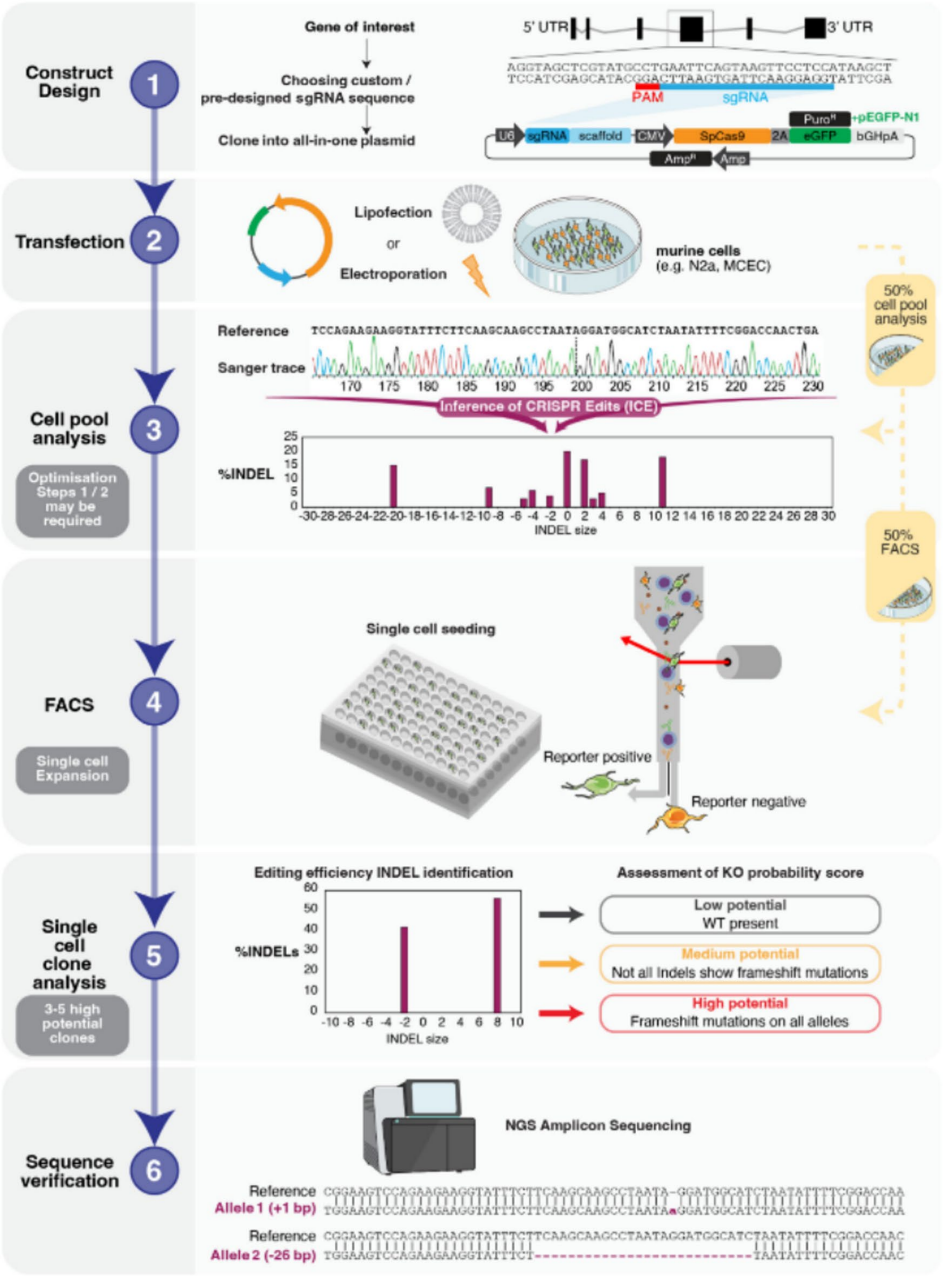
Stepwise workflow for generating single-cell knockout (KO) cell line (Hub et al. 2024)

The ideal solution to solve the above challenges is to integrate target genes efficiently and stably under various conditions, select effective integration sites, and applying targeted integration technology to the creation of industrial-grade cell lines. The gene-targeted integration technique mediated by the CRISPR/Cas9 system (Nidhi et al. 2021; Zhang et al. 2024) utilizes the nuclease activity of CRISPR/Cas9 to induce double-strand breaks at specific genomic regions. In the presence of donor vectors containing homologous arms and exogenous genes, the cell employs the homologous repair pathway to accurately insert the exogenous gene into a designated location within the genome (Katti et al. 2022; MacGillavry 2023). This targeted integration method enables the rapid and precise insertion of exogenous genes into the desired regions of the cell genome, achieving long-term stable high expression. There is an example to list the progress of CRISPR-based cell-line construction, refer to Fig. 1.

#### Mechanism of the CRISPR/Cas9 system

CRISPR, abbreviation of ‘Clustered Regularly Interspaced Short Palindromic Repeats’ (Zou et al. 2025), is a distinctive set of DNA sequences composed of a leader sequence, repeat sequences, and spacer sequences. The leader sequence is located at the forefront of the CRISPR region and acts as a promoter. The repeat sequences help stabilize the secondary structure of RNA. The spacer sequences are composed of DNA fragments captured by bacteria from the external environment and provide a defense mechanism against foreign gene invasion (Asmamaw Mengstie et al. 2024). The Cas genes are located near the CRISPR region or dispersed throughout the genome, encoding proteins that work in the synergy with CRISPR sequences. Therefore, these genes are collectively referred to as CRISPR-associated genes (Cas) (Zou et al. 2025; Asmamaw Mengstie et al. 2024; Li et al. 2020). Based on the types and functionality of Cas proteins, the CRISPR/Cas system is divided into three major classes: Class I, Class II, and Class III. Class II has become the most widely used set of Cas proteins (Yang et al. 2024).

The CRISPR/Cas9 system primarily includes the Cas9 protein and a single-guide RNA (sgRNA), where Cas9 is responsible for cutting the DNA double strand while the sgRNA provides targeting specificity. With the principle of base pairing, the Cas9 protein can target various DNA sites, thereby trigger cellular mechanisms of homologous recombination and non-homologous end joining repair. This allows the system to conduct editing or modifications at specified genetic loci, including gene knock-out, insertion, or alteration (Li et al. 2020; Jiang et al. 2019; Uddin et al. 2020).

#### Applications of CRISPR/Cas9 in the construction of RGA cell lines

The CRISPR/Cas9 technique has been successfully applied in the construction of various stable cell lines expressing reporter genes, including tumor cell lines and stem cell lines, etc. (Yang et al. 2024). The common method for constructing cell lines using CRISPR/Cas9 technology includes the following steps: (a) sgRNA design and synthesis: Design specific sgRNA based on the genomic sequence of the target cell to ensure efficient targeting to the integration site. (b) CRISPR/Cas9 vector construction: Clone the sgRNA sequence into the CRISPR/Cas9 expression vector, which can express the Cas9 protein and translate sgRNA in the cell, forming a complex. (c) Transfection: Introduce the CRISPR/Cas9 expression vector into the target cells through methods such as lipid-mediated transfection, electroporation, or other approaches. (d) Homologous recombination repair: Insert the reporter gene into the genomic integration site utilizing the cell homologous recombination repair mechanism to Wardyn et al. (2021).

The state-of-the-art CRISPR-derived base editors and prime editors have significantly advanced gene-editing technology, offering greater precision and flexibility. 

Table 5 lists a table comparing base editors and prime editors. Base editors can be used to correct point mutations in genes, generating stable cell lines with specific genetic backgrounds (Hryhorowicz et al. 2023). Prime editors offer greater flexibility and precision in genome editing. They can introduce precise insertions, deletions, and base substitutions without requiring donor DNA templates or generating double-strand breaks. PE4 (Cirincione et al. 2025) was used to generate stable cell lines with corrected mutations in the FANCC and VHL genes, providing promising tools for studying genetic diseases and developing gene therapies.

**Table 5:** The compare of base editors and prime editors (Zou et al. 2025)

Editor type	Editing Scope	Advantages	Disadvantages
Base editors	C → T, A → G, etc., point mutations	High editing efficiency, no double-strand breaks required, lower off-target effects	Limited editing scope, requiring the target site to be positioned relative to the PAM
Prime editors	All four transition point mutations, all eight transversion point mutations, insertions, deletions, etc	High precision, flexible editing scope, lower off-target effects, no requirement for the target site to be positioned relative to the PAM	Relatively complex editing process, relatively lower editing efficiency compared to base editors

The CRISPR/Cas9 technology has several advantages of high efficiency, low cost, and simple operation (Uddin et al. 2020). By screening “safe harbors” as integration sites, it ensures the genomic stability of recombinant cell lines, thereby guaranteeing the long-term stability during passages. The gene targeting integration technology mediated by CRISPR/Cas9 can be used to construct RGA cell lines and create stable transfected cell lines, with the advantages of high efficient, reliable, and stable. This provides strong support for the subsequent construction of subsequent reporter gene cell lines (Uddin et al. 2020).

Additionally, off-target is a critical issue in CRISPR-Cas9 gene editing. To mitigate off-target effects, various strategies have been developed, including gRNA modification and engineering, improved Cas variants (such as Cas9 nickase and SpCas9-HF1), off-target detection methods (Bowtie, BWN, CasOT), CRISPR delivery methods and other strategies etc. The article reports nucleic acids, prime editors, improved Cas variants, and optimized guide RNAs are key strategies for reducing off-target effects (Asmamaw Mengstie et al. 2024). The prime editing method showed higher efficiency and lower off-target effects compared to Cas9 nucleases.

#### CRISPR/Cas9-mediated site for exogenous gene integration

The application of CRISPR/Cas9 technology in targeted gene integration in mammalian cells is becoming increasingly widespread. Therefore, it is crucial to screen for a transcriptionally active and stable integration site that have minimal impact on cell lines. The main integration sites for exogenous genes in mammalian cells that have been reported includes: (a) ROSA26 site (Nakashiba et al. 2023): A widely used safe integration site in mice, often used for gene knockout and knock-in experiments to establish animal models with specific genetic backgrounds. (b) AAVS1 site (Gu et al. 2022): A known safe harbor site in the human genome, frequently used for gene editing. (c) CSN2 site (Smirnov et al. 2022): In cattle, the CRISPR/Cas9 technology has been applied to integrate the hFAD3 gene into the CSN2 site. d) NCAPG-LCORL site (Majeres et al. 2024): The CRISPR/Cas9 system has also been used to knock in gene expression cassettes like hFAD3 into the NCAPG-LCORL site of cattle. (e) Hprt site (Kawabe et al. 2018): HPRT encodes hypoxanthine phosphoribosyltransferase and is widely used as an integration site in mammalian cells, facilitating the stable high expression of target genes. (f) H2-Tw3 site (Miura et al. 2024): In certain mammalian cells, the H2-Tw3 site is also considered a viable integration site. (g) H11 site (Browning et al. 2020): The H11 site is another site commonly used for gene editing in mice, suitable for exogenous gene expression and establishing transgenic mouse models. The successful application of the above sites demonstrates the diversity and flexibility of CRISPR/Cas9 technology in mammalian genome editing, providing a powerful tool for constructing cell lines with reporter gene targeted integration (Hryhorowicz et al. 2023).

## Discussion

The rapid advancement of biologics has propelled the adoption of reporter gene assays (RGAs) as pivotal tools for evaluating biological activity. RGAs link reporter genes to intracellular signaling pathways, offering insights into drug effects on cell signaling. However, their utility depends on careful selection of reporter genes—prioritizing detectability, expression stability, and minimal physiological interference. Additionally, consistent cell line sourcing is critical for reproducibility, yet traditional random integration methods often yield unstable lines due to positional effects or promoter silencing.

CRISPR/Cas9 has revolutionized stable cell line construction by enabling precise gene editing. Unlike random integration, which risks unpredictable expression patterns, CRISPR allows targeted insertion into safe harbor loci (e.g., AAVS1, ROSA26), mitigating promoter silencing and positional variability. This precision ensures stable reporter expression, a key advantage over traditional methods. For instance, studies demonstrate that CRISPR-engineered lines exhibit reduced methylation-related silencing compared to randomly integrated clones. It is gradually becoming the ideal choice for constructing stable cell lines.

High-throughput screening (HTS)-compatible Cas9 variants and base editors enable rapid, scalable construction of reporter lines, this reduces developmental timelines while maintaining robustness. The compliance of regulatory guideline (such as ICH Q6B) is essential for clinical translation. CRISPR’s ability to ensure consistent integration sites and expression profiles aligns with requirements for product consistency and stability. The CRISPR-derived lines better meet validation criteria by eliminating random integration variability.

The reporter gene assay technology is a continuous evolving field, and the construction of stable cell lines is also making continuous progress. The harmonized guidelines for multiplex RGA validation will strengthen industry standards. CRISPR/Cas9 addresses historical limitations in RGA reliability, positioning RGAs as indispensable tools for biopharmaceutical development. With the emergence of new technologies and a deeper understanding of disease mechanisms, the reporter gene assays will play an even greater role in future drug evaluation and research. It will promote the standardization and regulation of quality systems for biologics and further development of the biopharmaceutical industry.

## Data Availability

Not applicable.
